# Craving under pressure: the interplay between hedonic hunger, mental health, and ultra-processed food consumption in shift-workers

**DOI:** 10.3389/fpubh.2026.1757016

**Published:** 2026-02-12

**Authors:** Elif Akin, Hatice Merve Bayram, Arda Ozturkcan

**Affiliations:** Department of Nutrition and Dietetics, Faculty of Health Sciences, Istanbul Gelisim University, Istanbul, Türkiye

**Keywords:** food choice, hedonic hunger, mental health, shift work, stress, ultra-processed foods

## Abstract

**Background:**

Shift-work is linked to irregular eating patterns and greater ultra-processed food (UPF) intake, potentially driven by hedonic hunger and psychological distress. This study aimed to examine the relationship between hedonic hunger, UPF consumption, and mental health among shift-working healthcare professionals.

**Methods:**

In this cross-sectional analytical observational study, 326 healthcare shift-workers (66.9% female) completed questionnaires including sociodemographic details, the Power of Food Scale (PFS-Tr), the Single-Item Food Choice Questionnaire (FCQ), the Depression Anxiety Stress Scale (DASS-21), and the short screening questionnaire for highly processed food consumption (sQ-HPF).

**Results:**

63.2% were categorized as high-level UPF consumers. PFS-Tr scores correlated positively with UPF intake, FCQ, depression, stress, and anxiety. Hedonic hunger was significantly associated with UPF consumption directly (β = 0.112) and indirectly through stress (β = 0.209).

**Conclusion:**

Hedonic hunger was associated with UPF intake in shift-workers through psychological distress and food motivation.

## Introduction

Following the Industrial Revolution, societies structured time around a work-life dichotomy, with conventional working hours dominating until the mid-20th century. However, technological and economic advancements led organizations to extend work schedules to 24/7 operations, resulting in nonstandard work arrangements such as shift work, night work, and weekend work (1). Shift work is generally defined as a work schedule where different teams rotate to cover most or all of the 24-hour day (2). Shift work is performed by around 15% to 20% of employees in Europe, 20% in the US, 6% to 32% in Asia and 8% in Poland (3). One of the sectors in which shift work is comon is the healthcare sector. Paramedics, nurses and doctors represent the medical professions (4).

The work system has an impact on both lifestyle and health. Research highlights shift work and night work as the most detrimental schedules, negatively affecting workers' health, family and social life, and overall organizational dynamics (4, 5). A widely debated issue is the impact on the health of people working different schedules (day, night or rotating) (6). People who work night shifts and rotating shifts often face greater health risks than their daytime counterparts. The health implications for people with different working patterns go beyond metabolic syndrome and risk factors such as obesity (7). Studies suggest that night shift work can elevate the risk of several health problems such as cardiovascular diseases, gastrointestinal disorders, psychological problems and even certain cancers, particularly breast and colorectal cancer (8–11). This increased risk is attributed to circadian disruption. Circadian disruption affects biological functions, alters hormone levels and leads to sleep disturbances (1).

Shift work is a critical factor influencing eating behaviors. Shift work has been found to be associated with higher food insecurity among US workers, indicating a lack of access to adequate nutrition (12). This is particularly relevant for healthcare workers who work non-standardized hours and face difficulties in maintaining a healthy diet due to disrupted dietary patterns. The literature has shown that shift workers, particularly night shift workers, tend to make unhealthy food choices, including greater consumption of sandwiches, cakes, potato chips, and biscuits, fried foods and lower intake of fruit and vegetables, compared to their day shift colleagues (13–15). Moreover, the timing of food intake is crucial. It has often been observed that night eating patterns that deviate from traditional mealtimes can lead to adverse physiological outcomes among night shift workers (16). Inappropriate meal times can affect the body's circadian rhythms. Furthermore, this dietary change can exacerbate health problems, as poor eating habits are associated with an increased risk of cardiometabolic disease among shift workers.

Additionally, the unique working conditions and stress associated with healthcare roles can further complicate nutritional status. A study reported that high stress levels can lead to unhealthy eating behaviors, contributing to deteriorating health markers like obesity (6). Similarly, Gao et al. identified a clear association between shift work and adverse health outcomes, reinforcing the notion that shift work affects nutrition and other health parameters (17).

In this context, hedonic hunger represents a key psychological mechanism through which occupational stress, circadian disruption, and constrained food environments may shape eating behavior among shift-working healthcare professionals. Hedonic hunger refers to a reward-driven tendency to consume palatable foods in the absence of physiological need, reflecting heightened sensitivity to food-related cues. In shift work, it is conceptualized as a context-sensitive disposition that may be amplified under conditions of stress, fatigue, and circadian disruption, rather than as a stable personality trait. These conditions are particularly relevant for healthcare professionals working shifts. There is a consensus that healthcare organizations should establish interventions that promote healthier eating habits among shift workers, but there are no evidence-based nutrition guidelines specifically tailored for shift workers. This is vital not only for individual health but also for the overall effectiveness of healthcare service delivery, as poor nutrition can hinder job performance and quality of patient care (18). Addressing nutritional deficiencies and promoting better health practices among shift-working healthcare workers is essential for improving their well being and working conditions. Therefore, the aim of this study was to evaluate the relationship between ultra-processed food (UPF) consumption, hedonic hunger and mental health among shift-working healthcare professionals.

## Methods

### Study design and participants

The study was designed as a cross-sectional analytical observational study, and was conducted on health professionals with shift work in Istanbul between 30 November 2024 and 30 March 2025.

The study population consisted of healthcare professionals working in shift-based systems in a tertiary healthcare hospital in Istanbul, Türkiye. Due to the absence of an institutional registry specifying the exact number of shift-working staff, the total population size was unknown. Accordingly, sample size adequacy was determined based on an a priori power analysis.

An online questionnaire including demographic characteristics, Power of Food Scale (PFS-Tr), Single-Item Food Choice Questionnaire (FCQ), Depression, Anxiety, Stress Scale (DASS-21), and a short screening questionnaire of ultra-processed food (UPF) consumption (sQ-HPF) was applied to the participants. Within the scope of this study, power analysis was performed using G^*^power to determine the sample size to examine the relationship between UPF consumption and hedonic hunger. Since correlation analysis was planned over a single group, a medium effect size (*r* = 0.3), 5% significance level (α = 0.05) and 95% power level (1 - β = 0.95) were used in the calculations. According to the results of the analyses, the minimum sample size required to analyse the relationship between these parameters was determined as 289 participants.

### Power of food scale

The Power of Food scale (PFS) was developed by Cappelleri et al. (19). Turkish validity and reliability of PFS (PFS-Tr) were conducted by Ülker et al. (20). The 5-point Likert-type scale (1: strongly disagree, 5: strongly agree) consists of 13 questions and 3 sub-dimensions: food available, food present, and food tasted. The total PFS-Tr score and the subscale score are obtained by summing the scores of the items and dividing the sum by the number of items.

### Depression, anxiety, stress scale

Depression, anxiety, and stress symptoms were assessed using the Depression, Anxiety, and Stress Scale Short Form (DASS-21) which was originally developed by Lovibond and Lovibond (21). Sariçam conducted an evaluation to assess the validity and reliability of the Turkish version of the DASS-21 (22). The questionnaire comprised seven items for each of the three scales (depression, anxiety and stress) designed to measure the negative emotional states of the scales. It employed a 4-point Likert-type scale, with the sum of the scores obtained multiplied by 2 and then evaluated in accordance with the severity rating index. For depression, the scores between 0 and 4 are defined as normal, 5–6 as mild depression, 7–10 as moderate depression, 11–13 as severe depression, and >13 as very severe depression. For anxiety, the scores between 0 and 3 are defined as normal, 4–5 as mild anxiety, 6–7 as moderate anxiety, 8–9 as severe anxiety, and > 9 as very severe anxiety. For stress, the scores between 0 and 7 are defined as normal, 8–9 as mild stress, 10–12 as moderate stress, 13–16 as severe stress, and > 16 as very severe stress (22).

### Single-item food choice questionnaire

The aim of the Single-Item Food Choice Questionnaire (FCQ) developed by Onwezen et al. (23) is to determine which factors are effective in individuals' food preferences. Turkish validity and reliability of the scale were performed by Haydaroglu et al. (24). The 7-point Likert-type scale consists of 11 items and the increase in the score indicates that the factor is effective in food selection.

### Short screening questionnaire of highly processed food consumption

The short screening questionnaire of highly processed food consumption scale (sQ-HPF) was developed by Martinez-Perez et al. (25). Turkish validity and reliability were performed by Erdogan Gömez et al. (26). The scale consists of 11 food groups and each food group is scored between 0 and 1 point, 6 scores and above is considered as high level UPF consumption.

### Statistical analysis

All statistical analyses were performed using IBM SPSS Statistics version 24.0. Descriptive statistics were calculated for all variables, and results were presented as means and standard deviations for continuous variables, and frequencies and percentages for categorical variables. Internal consistency reliability of all multi-item scales was assessed using Cronbach's alpha coefficients. The Kolmogorov-Smirnov test was used to test the normality of the variables. Group comparisons according to shift duration tertiles and gender were conducted using Kruskal-Wallis test, for continuous variables, and chi-square tests for categorical variables. Spearman correlation analyses were used to assess the associations between age, BMI, PFS-Tr scale and sub-scales, sQ-HPF scale, FCQ scale, and DASS-21 scale. Linear regression analyses were conducted to determine predictors of hedonic hunger and UPF consumption. Model fit was evaluated using the coefficient of determination (R^2^), representing the proportion of variance explained by each regression model. To investigate whether stress, depression, anxiety, and BMI mediated the relationship between hedonic hunger and UPF consumption, mediation analyses were conducted using the Baron and Kenny (1986) approach (27). These analyses were conducted in an exploratory manner to examine potential indirect associations and should not be interpreted as implying temporal or causal ordering among the variables. Each model tested three regression paths: (a) the effect of hedonic hunger on the mediator, (b) the effect of the mediator on UPF consumption while controlling for hedonic hunger, and (c) the total effect of hedonic hunger on UPF consumption. Partial mediation was supported when both the indirect and direct paths remained significant. The total effect (Path c) was derived from the same base regression model without mediators and therefore remained constant across mediation analyses. The significance threshold for all tests was set at *p* < 0.05.

## Results

The internal consistency of the measurement tools used in the study was evaluated using Cronbach's alpha coefficients. The PFS-Tr demonstrated excellent internal consistency (α = 0.946). Similarly, the DASS-21 showed very high reliability (α = 0.943). The FCQ exhibited excellent internal consistency (α = 0.923), while the sQ-HPF demonstrated acceptable internal consistency (α = 0.744).

Table 1 shows the demographic characteristics of the participants. A total of 326 (66.9% female) shift-working healthcare personnel completed the study. Most of the participants (79.8%) worked on night and morning and/or afternoon shifts, 15.6% on night and day shift and/or afternoon and 4.6% on night only.

**Table 1:** Demographic characteristics (*n*: 326).

**Variable**	**N**	**%**
**Gender**		
Male	108	33.1
Female	218	66.9
**Presence chronic disease**		
No	262	80.4
Yes	64	19.6
**Regular physical activity**		
No	241	73.9
Yes	85	26.1
**Regular sleep**		
No	256	78.5
Yes	70	21.5
**Type of shift**		
Night only	15	4.6
Night and day shift and/or afternoon	51	15.6
Night and morning and/or afternoon shifts	260	79.8
**Typical night shift duration**		
Less than 12 h	51	15.6
12–24 h	67	20.6
More than 24 h	208	63.8

Table 2 shows the demographic characteristics of continuous variables. The mean age of the participants was 30.00 ± 7.23 years, and the mean BMI was 24.87 ± 3.98 kg/m^2^. The mean shift time of participants was 19.57 ± 7.21 h.

**Table 2:** Descriptive statistics of continuous variables (*n*: 326).

**Variable**	**Mean ±SD**
Age	30.00 ± 7.23
Mean shift time	19.57 ± 7.21
Height (cm)	168.11 ± 8.16
Body weight (kg)	70.57 ± 13.92
BMI (kg/m^2^)	24.87 ± 3.98

BMI, body mass index.

Table 3 presents the distribution of hedonic hunger, food choice, and UPF consumption according to shift duration tertiles. Significant differences were found in sQ-HPF scores (*p*: 0.029), depression (*p* < 0.001), stress (*p*: 0.007), and anxiety (*p*: 0.002) between shift duration tertiles. Gender comparisons revealed significant differences in PFS-Tr total score (*p*: 0.003), its subscales, sQ-HPF (p: 0.005), FCQ (*p*: 0.048), and anxiety scores (*p*: 0.048). The sQ-HPF, stress and anxiety differences were observed between tertiles 1 and 2, while the depression difference was observed between tertiles 1 and 2 and 3. According to sQ-HPF classification, most of the participants were in the high level UPF consumption group (63.2%), while there was a significant difference between genders (*p*: 0.010).

**Table 3:** Distribution of hedonic hunger, food choice, and ultra-processed food consumption scores according to shift duration tertiles [median (25–75 percentiles)].

**Variable**	**Tertile 1 (Less than 12 h) (*n*: 51)**	**Tertile 2 (12–24 h) (*n*: 67)**	**Tertile 3 (>24 h) (*n*: 208)**	***P*^1^-value**	***P*^2^-value**
Food available	10.00 (8.00–15.00)	11.00 (7.00–14.00)	11.00 (8.00–14.00)	0.773	0.005^*^
Food present	13.00 (8.00–16.00)	12.00 (7.00–16.00)	13.00 (9.00–16.00)	0.205	0.020^*^
Food taste	18.00 (12.00–20.00)	17.00 (8.00–20.00)	17.00 (13.00–20.00)	0.373	0.002^*^
PFS-Tr total score	40.00 (30.00–52.00)	38.00 (21.00–50.00)	42.50 (32.00–50.00)	0.519	0.003^*^
sQ-HPF total score	6.00 (3.00–8.00)	7.00 (5.00–9.00)	6.00 (4.00–8.00)	0.029^*^	0.005^*^
**sQ-HPF classification**				0.093	0.010^*^
Low level UPF consumption	20 (39.2)	17 (25.4)	83 (39.9)		
High level UPF consumption	31 (60.8)	50 (74.6)	125 (60.1)		
FCQ total score	51.00 (30.00–60.00)	44.00 (28.00–60.00)	50.00 (37.25–60.00)	0.346	0.048^*^
Depression score	3.00 (1.00–7.00)	7.00 (5.00–11.00)	7.00 (3.00–9.00)	< 0.001^*^	0.916
**Depression classification**				0.005^*^	0.920
Normal	29 (56.9)	15 (22.4)	66 (31.7)		
Mild depression	9 (17.6)	13 (19.4)	30 (14.4)		
Moderate depression	7 (13.7)	22 (32.8)	68 (32.8)		
Severe depression	1 (2.0)	8 (11.9)	14 (6.7)		
Very severe depression	5 (9.8)	9 (13.5)	30 (14.4)		
Stress score	6.00 (3.00–8.00)	8.00 (5.00–11.00)	7.00 (5.00–11.00)	0.007^*^	0.434
**Stress classification**				0.007^*^	0.709
Normal	38 (74.6)	31 (46.3)	110 (52.9)		
Mild stress	4 (7.8)	7 (10.4)	32 (15.4)		
Moderate stress	3 (5.9)	15 (22.4)	35 (16.8)		
Severe stress	2 (3.9)	13 (19.4)	22 (10.6)		
Very severe stress	4 (7.8)	1 (1.5)	9 (4.3)		
Anxiety score	2.00 (1.00-5.00)	6.00 (3.00-8.00)	4.50 (2.00-7.00)	0.002^*^	0.048^*^
**Anxiety classification**				0.024^*^	0.475
Normal	31 (60.8)	20 (29.9)	91 (43.8)		
Mild anxiety	8 (15.7)	11 (16.4)	34 (16.3)		
Moderate anxiety	4 (7.8)	18 (26.9)	38 (18.3)		
Severe anxiety	5 (9.8)	4 (6.0)	18 (8.6)		
Very severe anxiety	3 (5.9)	14 (20.8)	27 (13.0)		

^*^p < 0.05 Data are expressed as median (25th−75th percentiles) due to non-normal distribution, *p*^1^ represents differences between shift duration tertiles assessed using the Kruskal–Wallis test, *p*^2^ represents differences between gender assessed using the Mann–Whitney U-test. PFS, the power of food scale; FCQ, Single-Item food choice questionnaire; sQ-HPF, short screening questionnaire of highly processed food consumption scale.

Age was weakly and negatively correlated with PFS-Tr total score (r: – 0.143, *p* < 0.05), food available (r: – 0.140, *p* < 0.05), sQ-HPF total score (r: – 0.246, *p* < 0.001) and anxiety score (r: – 0.155, *p* < 0.05) and weakly and positively correlated with BMI (*r*: 0.232, *p* < 0.001). BMI showed a weak positive correlation with food present (*r*: 0.150, *p* < 0.05) and sQ-HPF (*r*: 0.153, *p* < 0.05). PFS-Tr total score showed weak positive correlations with sQ-HPF total score (*r*: 0.172, *p* < 0.05), FCQ total score (*r*: 0.275, *p* < 0.001), depression score (*r*: 0.163, *p* < 0.05), stress score (*r*: 0.222, *p* < 0.001) and anxiety score (*r*: 0.157, *p* < 0.05). The sQ-HPF total score was weakly positively correlated with depression (*r*: 0.174, *p* < 0.05), stress (*r*: 0.168, *p* < 0.05) and anxiety scores (*r*: 0.179, *p* < 0.001) (Table 4).

**Table 4:** Correlation of age, BMI, PFS-Tr scale and sub-scales, sQ-HPF scale, FCQ scale, and DASS-21 scale.

**Variables**	**1**	**2**	**3**	**4**	**5**	**6**	**7**	**8**	**9**	**10**
1. Age										
2. BMI	0.232^**^									
3. Food available	–0.140^*^	0.096								
4. Food present	–0.099	0.150^*^	0.796^**^							
5. Food taste	–0.148^*^	0.016	0.762^**^	0.745^**^						
6. PFS-Tr total score	–0.143^*^	0.100	0.922^**^	0.917^**^	0.908^**^					
7. sQ-HPF total score	–0.246^**^	0.153^*^	0.170^*^	0.165^*^	0.1210	0.172^*^				
8. FCQ total score	–0.037	0.018	0.255^**^	0.237^**^	0.293^**^	0.275^**^	−0.087			
9. Depression score	–0.098	0.073	0.165^*^	0.165^*^	0.091	0.163^*^	0.174^*^	–0.075		
10. Stress score	–0.104	0.053	0.208^**^	0.214^**^	0.184^**^	0.222^**^	0.168^*^	–0.046	0.760^**^	
11. Anxiety score	–0.115^*^	–0.004	0.155^*^	0.143^*^	0.106	0.157^*^	0.179^**^	–0.100	0.743^**^	0.686^**^

^*^p < 0.05, ^**^p < 0.001. PFS, the power of food scale; FCQ, Single-Item food choice questionnaire; sQ-HPF, short screening questionnaire of highly processed food consumption scale.

Table 5 shows the stepwise linear regression predicting hedonic hunger. In Model 5, BMI (β: 0.456, *p*: 0.017), sQ-HPF (β: 0.538, p: 0.048), and stress (β: 0.609, p: 0.018) were significant predictors, accounting for 12.4% of the variance. Gender, and age were consistently significant predictors across all models.

**Table 5:** Linear regression analysis indicating the predictors of hedonic hunger.

**Model**	**Unstandardized coefficients**		**Standardized coefficients**						
	**B**	**SE**	**Beta**	**t**	**Sig**	**F**	**Sig**	**R**	*R* ^2^
1 (constant)	39.277	3.803		10.328	< 0.001^**^	8.399	< 0.001^**^	0.222	0.049
Age	–0.276	0.101	–0.150	–2.742	0.006^*^				
Gender	5.175	1.545	0.183	3.349	0.001^*^				
2 (constant)	24.119	6.047		3.989	< 0.001^**^	9.163	< 0.001^**^	0.280	0.079
Age	−0.343	0.101	−0.186	−3.376	0.001^*^				
Gender	6.488	1.578	0.229	4.111	< 0.001^**^				
BMI	0.601	0.188	0.180	3.195	0.002^*^				
3 (constant)	19.650	6.291		3.123	0.002^*^	8.384	< 0.001^**^	0.308	0.095
Age	–0.272	0.105	−0.147	−2.589	0.010^*^				
Gender	6.716	1.570	0.237	4.279	< 0.001^**^				
BMI	0.516	0.190	0.154	2.711	0.007^*^				
sQ-HPF	0.642	0.270	0.134	2.377	0.018^*^				
4 (constant)	9.208	6.213		1.482	0.139	14.773	< 0.001^**^	0.433	0.188
Age	−0.205	0.100	−0.111	−2.043	0.042^*^				
Gender	5.696	1.499	0.201	3.800	< 0.001^**^				
BMI	0.415	0.181	0.124	2.288	0.023^*^				
sQ-HPF	0.768	0.257	0.160	2.988	0.003^*^				
FCQ	0.252	0.042	0.309	6.050	< 0.001^**^				
5 (constant)	17.673	6.250		2.828	0.005^*^	6.436	< 0.001^**^	0.352	0.124
Age	−0.244	0.104	−0.132	−2.336	0.020^*^				
Gender	6.428	1.571	0.227	4.092	< 0.001^**^				
BMI	0.456	0.190	0.136	2.402	0.017^*^				
sQ-HPF	0.538	0.271	0.112	1.985	0.048^*^				
Depression	0.080	0.251	0.030	0.317	0.751				
Stress	0.609	0.257	0.209	2.373	0.018^*^				
Anxiety	−0.289	0.263	−0.094	−1.101	0.272				

^*^p < 0.05, ^**^p < 0.001. R represents the multiple correlation coefficient, and R^2^ represents the proportion of variance explained by the model. PFS, the power of food scale; FCQ, Single-Item food choice questionnaire; sQ-HPF, short screening questionnaire of highly processed food consumption scale.

Table 6 presents regression results for sQ-HPF. Model 5 indicates BMI (β: 0.111, *p*: 0.005) and PFS-Tr (β: 0.030, *p*: 0.013) as significant predictors. The model explained 16.1% of the variance. Although stress was added in the final model, it did not remain a significant independent predictor.

**Table 6:** Linear regression analysis indicating the predictors of sQ-HPF scale.

**Model**	**Unstandardized coefficients**		**Standardized coefficient**						
	**B**	**SE**	**Beta**	**t**	**Sig**	**F**	**Sig**	**R**	*R* ^2^
1 (constant)	10.328	0.782		13.213	< 0.001^**^	13.986	< 0.001^**^	0.282	0.080
Age	–0.095	0.021	−0.248	−4.605	< 0.001^**^				
Gender	−0.648	0.318	−0.110	−2.039	0.042^*^				
2 (constant)	6.966	1.240		5.619	< 0.001^**^	13.624	< 0.001^**^	0.336	0.113
Age	−0.110	0.021	−0.286	−5.290	< 0.001^**^				
Gender	−0.357	0.324	−0.060	−1.102	0.271				
BMI	0.133	0.039	0.191	3.457	0.001^*^				
3 (constant)	6.316	1.261		5.009	< 0.001^**^			0.358	0.128
Age	−0.101	0.021	−0.262	−4.797	< 0.001^**^				
Gender	−0.531	0.330	−0.090	−1.613	0.108				
BMI	0.117	0.039	0.168	3.011	0.003^*^				
PFS-Tr	0.027	0.011	0.129	2.377	0.018^*^				
4 (constant)	6.937	1.281		5.417	< 0.001^**^	10.626	< 0.001^**^	0.377	0.142
Age	−0.103	0.021	−0.266	−4.909	< 0.001^**^				
Gender	−0.494	0.328	−0.084	−1.508	0.132				
BMI	0.119	0.039	0.171	3.087	0.002^*^				
PFS-Tr	0.035	0.012	0.169	2.988	0.003^*^				
FCQ	−0.022	0.009	−0.127	−2.319	0.021^*^				
5 (constant)	6.593	1.282		5.144	< 0.001^**^	7.618	< 0.001^**^	0.402	0.161
Age	−0.096	0.021	−0.249	−4.573	< 0.001^**^				
Gender	−0.597	0329	−0.101	−1.816	0.070				
BMI	0.111	0.039	0.159	2.861	0.005^*^				
PFS-Tr	0.030	0.012	0.143	2.486	0.013^*^				
FCQ	−0.018	0.009	−0.104	−1.871	0.062				
Depression	−0.015	0.051	−0.026	−0.288	0.774				
Stress	0.031	0.053	0.051	0.587	0.557				
Anxiety	0.079	0.054	0.122	1.461	0.145				

^*^p < 0.05, ^**^p < 0.001. R represents the multiple correlation coefficient, and R^2^ represents the proportion of variance explained by the model. PFS, the power of food scale; FCQ, Single-Item food choice questionnaire; sQ-HPF, short screening questionnaire of highly processed food consumption scale.

**Figure 1:**
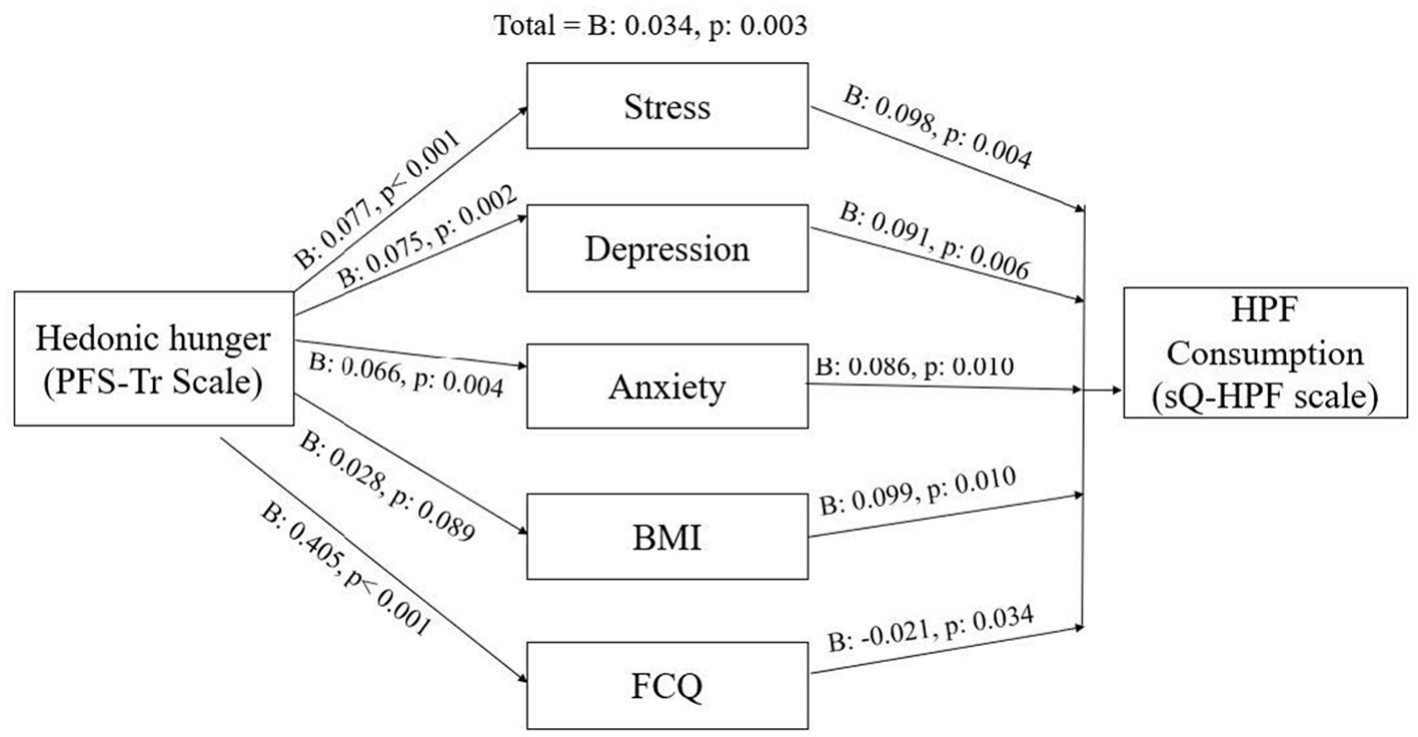
Exploratory mediation model illustrating statistical associations among hedonic hunger, psychological variables (depression, stress, anxiety), food choice factors (FCQ), BMI, and ultra-processed food consumption.

A series of mediation analyses were conducted to examine whether stress, depression, anxiety, and BMI mediated the relationship between hedonic hunger and UPF consumption. Hedonic hunger significantly predicted all three psychological variables—stress (*B*: 0.077, *p* < 0.001), depression (*B*: 0.075, *p*: 0.002), and anxiety (*B*: 0.066, *p*: 0.004)—each of which in turn significantly predicted UPF consumption: stress (*B*: 0.098, *p*: 0.004), depression (*B*: 0.091, *p*: 0.006), and anxiety (*B*: 0.086, *p*: 0.010). In all three models, hedonic hunger remained a significant predictor of UPF consumption when the mediator was included (B values ranged from 0.026 to 0.028), indicating partial mediation. In contrast, although BMI was a significant predictor of UPF (*B*: 0.099, *p*: 0.010), it was not significantly predicted by hedonic hunger (*B*: 0.028, *p*: 0.089); therefore, BMI did not meet the criteria for mediation. Furthermore, the total FCQ score was examined as a mediator. Hedonic hunger significantly predicted the FCQ total score (*B*: 0.405, *p* < 0.001), which in turn negatively predicted UPF consumption (*B*: −0.021, *p*: 0.034). When both variables were included, hedonic hunger remained a significant predictor (*B*: 0.034, *p*: 0.003), indicating a partial mediation with an inverse relationship. The total effect value remained constant across all models, as it was derived from the same base regression model without mediators, confirming the consistency and validity of the analysis (Figure 1). These findings reflect statistical indirect associations observed within the sample and do not establish causal directionality between hedonic hunger, psychological variables, BMI, and UPF consumption.

## Discussion

To the best of our knowledge, this is the first study to comprehensively evaluate the relationship between hedonic hunger, level of UPF consumption, and mental health parameters among shift-working healthcare professionals. Rather than novelty by population alone, the present study contributes by empirically demonstrating how hedonic hunger was statistically linked via psychological stress to influence UPF consumption within a circadian-disrupted occupational context. Although the study does not include a non–shift-working comparison group, its primary objective was not to contrast occupational groups, but to examine the internal psychological pathways linking hedonic hunger to UPF consumption within a population already known to be metabolically and psychologically vulnerable. Shift work constitutes a high-risk context characterized by circadian misalignment, elevated stress, and constrained food environments; therefore, identifying mechanisms within this group has direct clinical and occupational relevance. The findings revealed that 63.2% of participants were classified as high level UPF consumption, with hedonic hunger emerging as a significant predictor of UPF intake through both direct and indirect pathways—particularly via stress and FCQ. Furthermore, participants working longer shifts reported significantly higher levels of UPF consumption, depression, anxiety, and stress. These results underscore the multifactorial nature of dietary behaviors in shift workers, shaped not only by occupational demands but also by underlying psychological mechanisms, highlighting an urgent need for integrated nutritional and mental health strategies in healthcare settings.

Psychological distress is notably prevalent among healthcare shift workers, especially those on night shifts. Studies have shown that this group is more vulnerable to irritability, stress, depression, and anxiety, largely due to factors such as job insecurity, sleep disruption, and sustained exposure to infectious environments (28–30). Consistent with this evidence, our study found that participants working longer shifts (>24 h) reported significantly higher depression, anxiety, and stress scores compared to those with shorter shift durations. Moreover, gender-based comparisons revealed that female shift workers experienced significantly higher anxiety levels, reinforcing the notion of a gendered vulnerability in mental health outcomes, which is in line with previous findings identifying younger female non-physician staff as a particularly at-risk group (29). Beyond descriptive trends, our regression and mediation analyses demonstrated that stress significantly mediated the relationship between hedonic hunger and UPF consumption, underlining the indirect impact of psychological burden on dietary behavior. Although depression and anxiety were also tested as potential mediators, their effects were less pronounced, suggesting that stress may serve as the primary psychological conduit through which emotional drivers translate into unhealthy food choices. These findings further support the view that mental health is not only a personal well being concern but also a determinant of health behavior, particularly in demanding shift-based healthcare roles. Furthermore, the mediation findings should be interpreted with caution. Although the analytical models were specified with hedonic hunger as the independent variable, this does not imply that hedonic hunger causally precedes psychological distress or BMI. Dominant models of eating behavior emphasize that psychological distress and environmental stressors often shape eating patterns rather than emerge from them. In line with this literature, the present findings are best viewed as exploratory and hypothesis-generating, highlighting potential psychological pathways that warrant further examination in longitudinal or experimental designs. It should also be acknowledged that the observed effect sizes were small and the explained variance was modest. This is consistent with the multifactorial nature of eating behavior, suggesting that hedonic hunger and psychological variables represent partial contributors rather than dominant determinants of UPF consumption. Accordingly, the findings should be interpreted as indicating meaningful but limited associations within a complex behavioral system.

Shift workers, particularly nurses and emergency medical personnel, frequently experience circadian rhythm disruptions due to irregular work schedules, which in turn contribute to erratic eating patterns and a heightened susceptibility to hedonic hunger, a form of appetite driven by the rewarding aspects of food rather than physiological necessity. Previous research has linked elevated stress, anxiety, and depression levels in female shift workers to maladaptive eating behaviors, such as emotional and external eating, as well as restrained eating related to BMI increases (31). Supporting these findings, our study revealed that hedonic hunger was significantly associated with UPF consumption, and that stress partially mediated this relationship. Furthermore, female participants had significantly higher levels of hedonic hunger, anxiety, and UPF consumption, suggesting a gendered vulnerability consistent with earlier literature.

In our sample, 63.2% of participants were classified as high-level of UPF consumers, and those working longer shifts (>24 h) exhibited significantly higher UPF intake as well as elevated depression, anxiety, and stress scores. These patterns align with previous evidence indicating that shift workers often compensate for fatigue and psychological strain by skipping meals, snacking more frequently, and opting for energy-dense, nutritionally poor foods (15, 32, 33). These behaviors are further reinforced by environmental factors—such as limited access to healthy food options during night shifts—which increase reliance on hedonic, convenience-based eating (16). Additionally, our findings showed that FCQ was negatively associated with UPF intake, suggesting that individuals who place greater emphasis on deliberate food choice considerations may be less likely to translate hedonic hunger into higher ultra-processed food consumption.

Circadian misalignment may be associated with hormonal fluctuations, particularly in cortisol and ghrelin levels, which disrupt normal hunger cues and amplify hedonic drives toward UPF consumption (34). This misalignment has also been implicated in the development of metabolic syndrome and obesity among shift workers (35, 36). Within this context, our study provides novel evidence that hedonic hunger functions not only as a direct predictor of UPF consumption but also as a psychological mechanism operating through stress, mood disturbances, and motivational drivers, highlighting the multifaceted vulnerability of healthcare shift workers to unhealthy dietary behaviors.

### Clinical and policy implications

From a clinical and policy perspective, identifying stress as the primary mediator between hedonic hunger and UPF consumption has significant implications for shift-working healthcare professionals. Workplace interventions targeting stress management, such as structured break times, mental health screenings, and psychosocial support programmes, may indirectly contribute to healthy eating behaviors by reducing stress-induced hedonic eating. Furthermore, improving the food environment in healthcare settings by increasing access to affordable, minimally processed, and nutrient-dense foods, particularly during night shifts, may reduce dependence on consumption of UPFs. At the policy level, developing specialized occupational nutrition guidelines and corporate food policies for shift workers could be an effective strategy to reduce long-term cardiometabolic and mental health risks associated with shift work.

This study has severa l limitations that should be acknowledged. First, the cross-sectional design precludes any conclusions regarding causality between hedonic hunger, UPF consumption, and psychological outcomes. Longitudinal or experimental studies would be needed to establish the directionality of these associations. Second, data were collected via self-reported online questionnaires, which may be subject to reporting bias, including social desirability and recall bias. Third, the study sample, although sufficiently powered, was limited to healthcare professionals working in Istanbul and may not fully represent shift-working populations in other geographic or occupational contexts. Despite these limitations, the study also presents notable strengths. To our knowledge, it is the first to examine the interrelationships between hedonic hunger, UPF consumption, and mental health parameters in shift-working healthcare personnel using a mediation model. The use of validated and reliable measurement tools—including the PFS-Tr, sQ-HPF, FCQ, and DASS-21—enhances the methodological robustness of the study. Moreover, the comprehensive statistical analysis, including linear regression and mediation testing, offers valuable insight into the psychological and behavioral mechanisms underlying poor dietary habits among shift workers. These strengths support the relevance of our findings for informing future research, workplace health policy, and targeted interventions in clinical settings.

## Conclusion

This study highlights the significant interplay between hedonic hunger, UPF consumption, and psychological distress among shift-working healthcare personnel. The findings indicate that hedonic hunger is a key factor influencing dietary behaviors in this population, both directly and indirectly through elevated stress levels and food choices. A substantial proportion of participants exhibited high levels of UPF intake, particularly those working extended shifts and female staff members, who also reported higher anxiety and hedonic hunger scores. These results highlighted the urgent need for integrated interventions addressing both nutritional and mental health challenges in shift-working healthcare personnel. Tailored workplace policies, improved food environments, and psychological support services may offer effective strategies for mitigating the negative health impacts associated with shift work.

This study has certain limitations, including its cross-sectional design, reliance on self-reported measures, and the restriction of the sample to healthcare shift workers from a single institution, which may limit causal inference and generalizability. Future studies using longitudinal designs, objective dietary assessments, and intervention-based approaches are warranted to further elucidate the mechanisms linking hedonic hunger, psychological distress, and ultra-processed food consumption.

## Data Availability

The raw data supporting the conclusions of this article will be made available by the authors, without undue reservation.
